# Efficiency of transcription and translation of cell-free protein synthesis systems in cell-sized lipid vesicles with changing lipid composition determined by fluorescence measurements

**DOI:** 10.1038/s41598-024-53135-8

**Published:** 2024-02-03

**Authors:** Akari Miwa, Masatoshi Wakamori, Tetsuro Ariyoshi, Yasushi Okada, Mikako Shirouzu, Takashi Umehara, Koki Kamiya

**Affiliations:** 1 Division of Molecular Science, Graduate School of Science and Technology, Gunma University, 1-5-1 Tenjin-Cho, Kiryu, Gunma 376-8515 Japan; 2 Laboratory for Epigenetics Drug Discovery, RIKEN Center for Biosystems Dynamics Research, 1-7-22 Suehiro-Cho, Tsurumi-Ku, Yokohama, 230-0045 Japan; 3 Laboratory for Cell Polarity Regulation, RIKEN Center for Biosystems Dynamics Research, 6-2-3 Furue-Dai, Suita, Osaka 565-0874 Japan; 4 Department of Cell Biology, Graduate School of Medicine, and International Research Center for Neurointelligence (WPI-IRCN), the University of Tokyo, 7-3-1 Hongo, Bunkyo-Ku, Tokyo, 113-0033 Japan; 5 Department of Physics and Universal Biology Institute (UBI), Graduate School of Science, the University of Tokyo, 7-3-1 Hongo, Bunkyo-Ku, Tokyo, 113-0033 Japan; 6 Laboratory for Protein Functional and Structural Biology, RIKEN Center for Biosystems Dynamics Research, 1-7-22 Suehiro-Cho, Tsurumi-Ku, Yokohama, 230-0045 Japan

**Keywords:** Biochemistry, Membrane biophysics, Biomaterials

## Abstract

To develop artificial cell models that mimic living cells, cell-sized lipid vesicles encapsulating cell-free protein synthesis (CFPS) systems are useful for protein expressions or artificial gene circuits for vesicle–vesicle communications. Therefore, investigating the transcriptional and translational properties of CFPS systems in lipid vesicles is important for maximizing the synthesis and functions of proteins. Although transcription and translation using CFPS systems inside lipid vesicles are more important than that outside lipid vesicles, the former processes are not investigated by changing the lipid composition of lipid vesicles. Herein, we investigated changes in transcription and translation using CFPS systems inside giant lipid vesicles (approximately 5–20 μm in diameter) caused by changing the lipid composition of lipid vesicles containing neutral, positively, and negatively charged lipids. After incubating for 30 min, 1 h, 2 h, and 4 h, the transcriptional and translational activities in these lipid vesicles were determined by detecting the fluorescence intensities of the fluorogenic RNA aptamer on the 3′-untranslated region of mRNA (transcription) and the fluorescent protein sfCherry (translation), respectively. The results revealed that transcriptional and translational activities in a lipid vesicle containing positively charged lipids were high when the protein was synthesized using the CFPS system inside the lipid vesicle. Thus, the present study provides an experimental basis for constructing complex artificial cell models using bottom-up approaches.

## Introduction

CFPS systems can be powerful platforms for research in biochemistry^1,2^ nanotechnology^3^, biophysics^4^, synthetic biology^5,6^, and biomedicine^7^ because they can synthesize diverse proteins in vitro, including enzymes^8^, membrane proteins^9–11^, and cytotoxic proteins^12^. CFPS systems can be encapsulated into lipid vesicles and artificial cell models can be constructed using a bottom-up approach using such lipid vesicles to facilitate functions and interactions of proteins synthesized using these CFPS systems into the lipid vesicles^13–18^. Furthermore, a more complex system can facilitate the control of transcription and translation stimulated by additional reagents and communication between two lipid vesicles or lipid vesicles and living cells regulated by an artificial gene circuit^19–22^. Studies on the transcriptional and translational properties of lipid-vesicle-encapsulated CFPS systems have contributed to maximizing protein synthesis and functions. In a study, real-time transcription and translation using CFPS systems outside^23^ and inside lipid vesicles^24–27^ were observed using various methods, including the fluorescence intensities of the fluorescent probe-binding messenger RNA (mRNA) and fluorescence protein for observing transcription and translation, respectively. Studies on protein synthesis outside lipid vesicles that are approximately several hundred nm in diameter have investigated transcription and translation using CFPS systems by changing template DNA concentration and lipid composition of the lipid vesciels^23,28^. Positively or negatively charged lipids in such lipid vesicles affect the regulation of transcription and translation performed using CFPS systems. Conversely, some studies on protein synthesis inside lipid vesicles that are approximately 5–20 μm in diameter have investigated transcription and translation using CFPS systems by changing template DNA concentration, and neutral or negatively charged lipids^13,25,29–33^. Transcription and translation using CFPS systems inside lipid vesicles that mimic living cells are more important than that outside lipid vesicles. However, the changes of transcription and translation using CFPS systems inside lipid vesicles have not been simultaneously investigated by changing the lipid composition of the lipid vesicles containing positively charged lipids.

**Figure 1:**
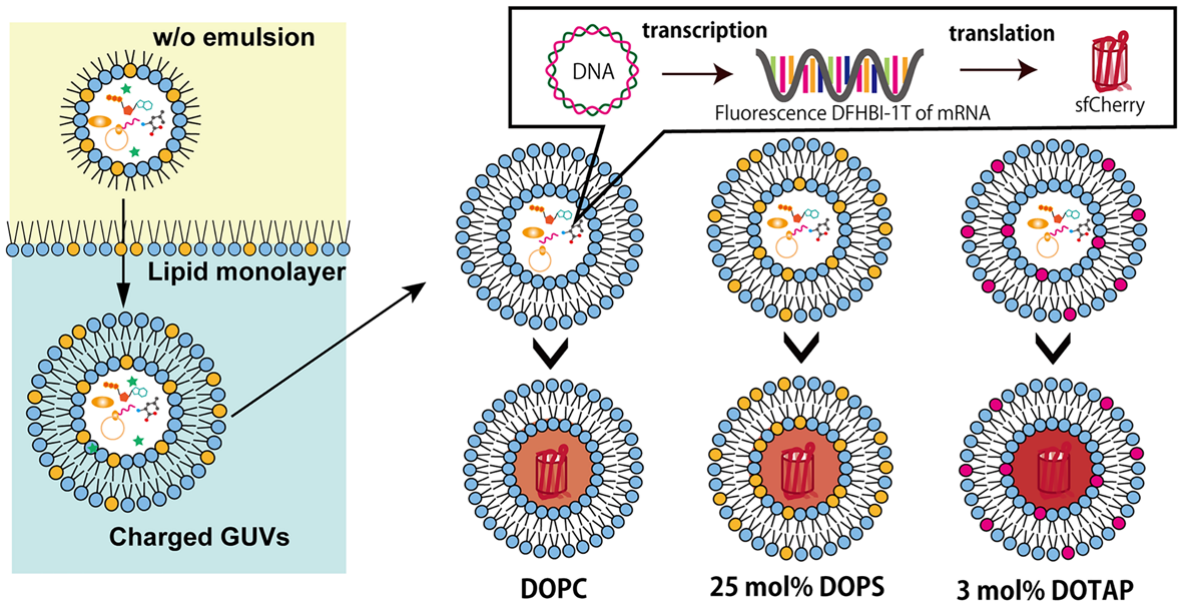
Schematic representation of giant lipid vesicle formation containing CFPS systems and investigation of transcription and translation in giant lipid vesicle containing neutral, negatively, or positively-charged lipid.

Herein, we investigated whether changing the lipid composition of lipid vesicles containing neutral, positively, and negatively charged lipids caused alterations in transcription and translation using CFPS systems inside giant lipid vesicles (approximately 5–20 μm in diameter) (Fig. 1). The transcription and translation dynamics of CFPS systems were monitored using a plasmid DNA (pDNA) construct containing the coding sequence for a red fluorescent protein sfCherry followed by a tandem array of RNA aptamer sequences that specifically binds to a green fluorogenic ligand, 3,5-difluoro-4-hydroxybenzylidene-imidazolinone (DFHBI-1T). First, we quantified the concentration of pDNA encapsulated in the three types of lipid vesicles. Next, we observed the fluorescence intensities of DFHBI-1T-binding mRNA and sfCherry after incubating the vesicles with varying concentrations of pDNA for 30 min, 1, 2, and 4 h. The green fluorescence-emitting DFHBI-1T indicated a transcription signal, whereas the red fluorescence-emitting sfCherry indicated a translation signal.

## Results and discussion

### Encapsulation of pDNA into the giant lipid vesicles

**Figure 2:**
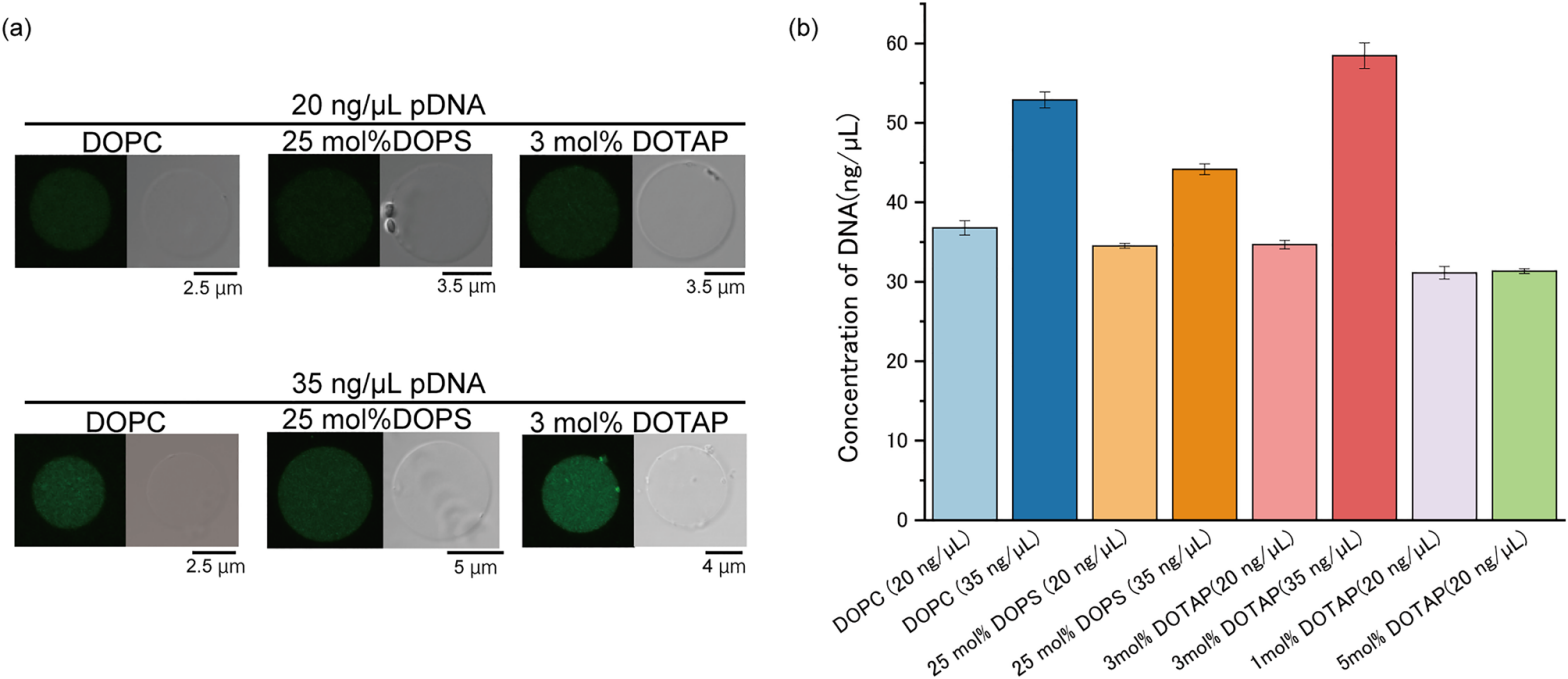
(**a**) Typical confocal images of the fluorescence intensities of oxazole yellow homodimer (YOYO-1)-conjugated sfCherry-producing plasmids under each membrane condition. (**b**) YOYO-1 analysis of plasmid DNA concentration. 1,2-dioleoyl-*sn*-glycero-3-phosphocholine (DOPC)-containing lipid vesicle (blue), three independent experiments; 25 mol% 1,2-dioleoyl-*sn*-glycero-3-phospho-l-serine (DOPS)-containing lipid vesicle (orange), three independent experiments; and 3 mol% 1,2-dioleoyloxy-3-(trimethylammonio)propane (DOTAP)-containing lipid vesicle (pink), three independent experiments; and 1-mol%-DOTAP-containing lipid vesicle (purple), three independent experiments; and 5-mol%-DOTAP-containing lipid vesicle (green), three independent experiments. Error bars indicate standard deviation (SD). All data of YOYO-1 fluorescence intensities was shown in Fig. S1.

To determine the concentration of encapsulated pDNA in the three types of giant lipid vesicle, namely 1,2-Dioleoyl-*sn*-glycero-3-phosphocholine (DOPC), DOPC/1,2-dioleoyl-*sn*-glycero-3-phospho-l-serine (DOPS) (3:1 molar ratio), and DOPC/1,2-dioleoyl-3-trimethylammonium-propane (DOTAP) (97:3 molar ratio), the giant lipid vesicles including the cell-free synthesis system without DFHBI-1T and YOYO-1-conjugated plasmid DNA were generated via the droplet transfer method. When the giant lipid vesicles were prepared using 20 ng/μL of pDNA, approximately 35 ng/μL of pDNA was encapsulated by all the vesicles. The concentration of encapsulated pDNA was not significantly different in the three types of giant lipid vesicles (Fig. 2a,b and Fig. S1a,c–g). Moreover, the encapsulated pDNA concentration in the giant lipid vesicles comprising DOPC/DOTAP (95:5 molar ratio and 99:1 molar ratio) was determined. The results showed that this concentration was not significantly different from those of the other components of the giant lipid vesicles (Fig. 2b). The fluorescence of YOYO-1 was not observed on the membranes of all giant lipid vesicles comprising DOPC/DOTAP (95:5 molar ratio), as shown in Fig. S1a. An electrostatic interaction between pDNA and membranes containing positively charged lipids (DOTAP) at 1–5 mol% did not occur because the CFPS system solution was present in the inner phase of the giant lipid vesicles.

When the giant lipid vesicles were prepared using 35 ng/μL of pDNA, the encapsulation concentration was higher compared with those prepared with 20 ng/μL of pDNA. The encapsulation concentration of pDNA in DOPC/DOTAP-containing vesicles was the highest among all. The encapsulated concentration varied among the three types of vesicles. The negatively charged pDNA is more efficiently encapsulated to the vesicles containing DOTAP, a positively charged phospholipid (Fig. 2a,b and Fig. S1h–j).

### Observation of transcription and translation in the giant lipid vesicles

**Figure 3:**
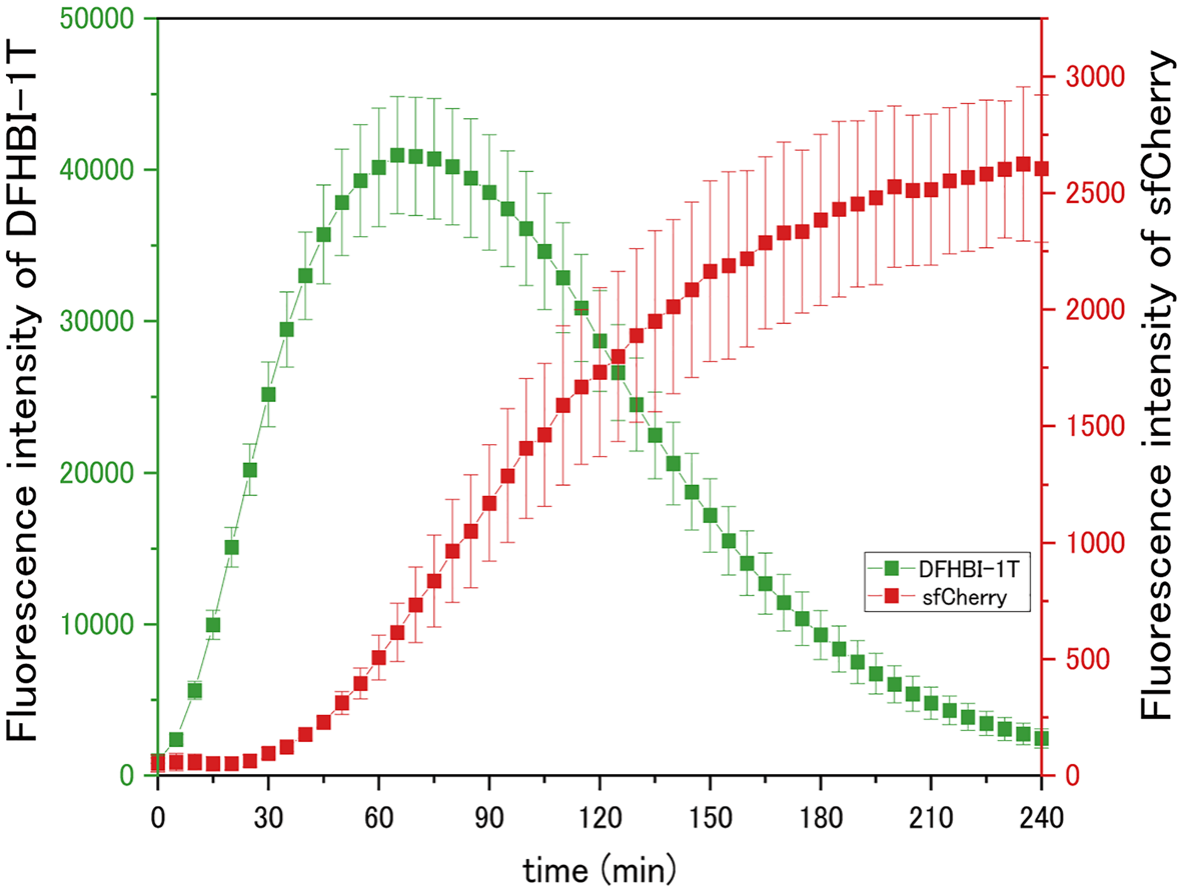
Time-dependent change on fluorescence intensity of DFHBI-1T and sfCherry in bulk solution. Mean values (three independent experiments) of fluorescence intensity of DFHBI-1T (Green Square),), fluorescence intensity of sfCherry (Red Square). Data are expressed as the mean ± SD.

To observe the transcription and translation of *E. Coli* extract S30 system in the vesicles, the mRNAs encoding sfCherry followed by a tandem array of RNA aptamers binding to DFHBI-1T were synthesized using a cell-free synthesis system. The fluorescence intensity of DFHBI-1T correlates with the amount of synthesized mRNA, and thus can be defined as the transcriptional efficacy. The translation was confirmed based on the fluorescence intensity of sfCherry. First, changes in each fluorescence intensity caused by the transcription and translation were observed using a microplate reader. The maximum fluorescence intensity of DFHBI-1T was observed after 60 min incubation, which decreased over time. In contrast, the maximum fluorescence of sfCherry was observed after 240 min incubation (Fig. 3). Hence, the activation of transcription and translation was detected based on these increasing and decreasing fluorescence intensities. On the other hand, although the activation of the translation of PUREfrex (CFPS system of complete reconstruction) was later than that of *E. coli* extract S30 system, the decrease in fluorescence intensity of DFHBI-1T was not observed for 4 h (Fig. S2a). Therefore, the decrease in fluorescence intensity of DFHBI-1T in *E. coli* extract S30 system was caused by a digestion of mRNA owing to *E. coli* extract S30 solution that is not completely free of nuclease.

**Figure 4:**
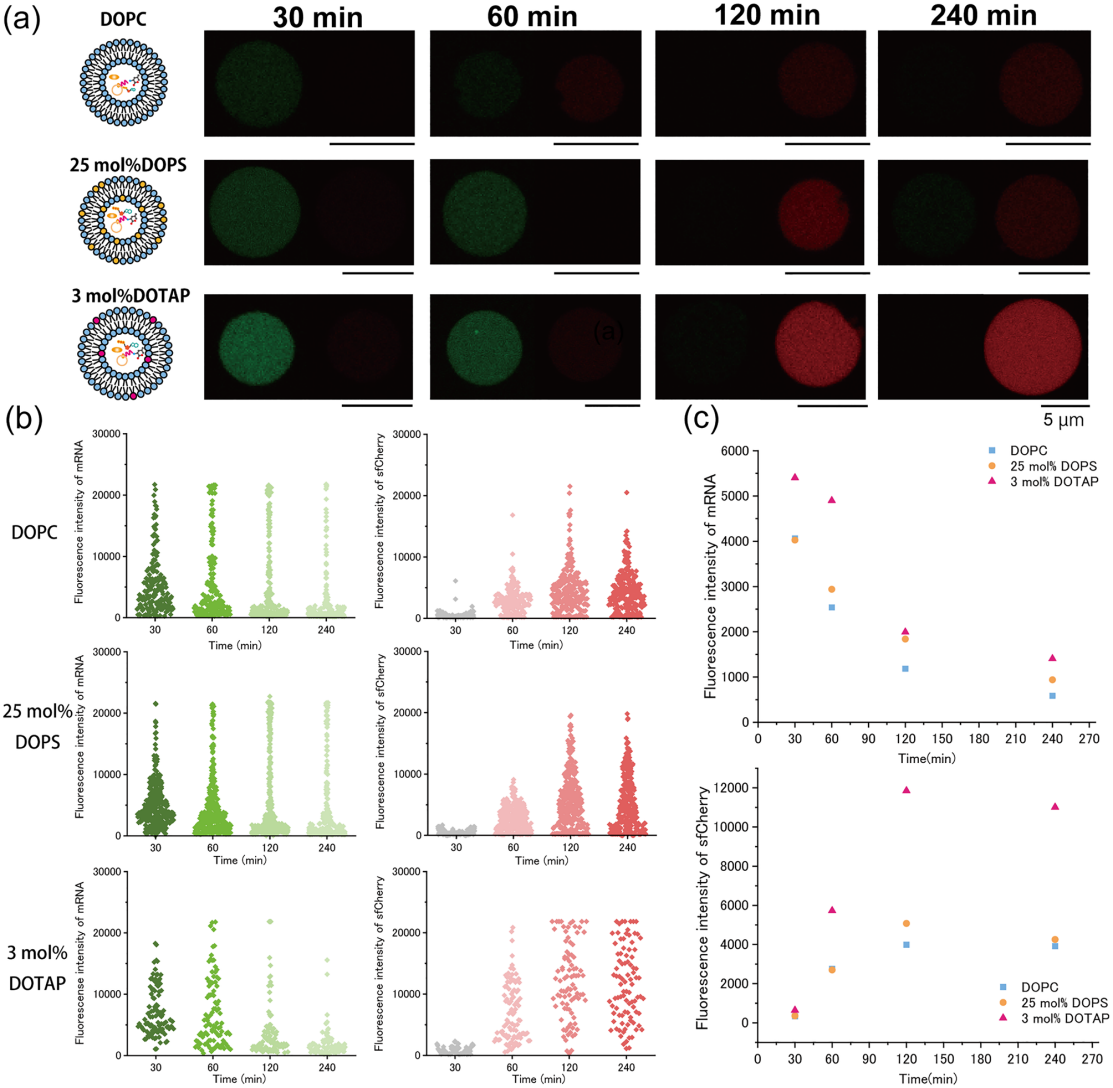
Comparison of transcription and translation inside giant lipid vesicles containing 20 ng/μL (final concentration) of plasmid DNA. (**a**) Typical confocal images of the fluorescence intensity change inside the lipid vesicles containing DOPC, DOPC/DOPS (75:25 molar ratio), or DOPC/DOTAP (97:3 molar ratio) on both leaflets. (**b**) DFHBI-1T fluorescence inside GUVs with DOPC, 25-mol%-DOPS or 3-mol%-DOTAP membrane in response to time changing shown in left (green). sfCherry fluorescence of GUVs with DOPC, 25-mol%-DOPS or 3-mol%-DOTAP membrane in response to time changing shown in right (red). DOPC vesicles; 30 min (n = 184 (184 vesicles), N = 2 (two independent experiments)), 60 min (n = 191, N = 2), 120 min (n = 259, N = 2), and 240 min (n = 247, N = 2). 25-mol%-DOPS vesicles; 30 min (n = 318, N = 3), 60 min (n = 322, N = 3), 120 min (n = 328, N = 3), and 240 min (n = 307, N = 3). 3-mol%-DOTAP vesicles; 30 min (n = 104, N = 5), 60 min (n = 102, N = 5), 120 min (n = 90, N = 5), and 240 min (n = 92, N = 5). (**c**) Time-dependent changes in fluorescence intensity of DFHBI-1T bound to messenger RNAs in the lipid vesicles. Comparison of the median value of DOPC-containing lipid vesicle (blue), 25-mol%-DOPS-containing lipid vesicle (orange), and 3-mol%-DOTAP-containing lipid vesicle (pink). Time-dependent changes in fluorescence intensity of sfCherry in the lipid vesicles. Comparison of the median value of DOPC-containing lipid vesicle (blue), 25-mol%-DOPS-containing lipid vesicle (orange), and 3-mol%-DOTAP-containing lipid vesicle (pink).

The activation of transcription and translation in the DOPC-containing vesicles was observed using CLMS. Due to the rapid initiation of transcription (starting from approximately 5 min), the observation of the initiation reaction was difficult and took 15 min to prepare the solutions of giant lipid vesicles containing cell-free transcription and translation system. Herein, the vesicles were observed after incubation for 30 min, 1 h, 2 h, and 4 h. For DOPC-containing vesicles, the fluorescence intensity of DFHBI-1T was the maximum at 30 min, which then decreased gradually. The DFHBI-1T fluorescence was rarely observed after 120-min incubation. In contrast, the sfCherry fluorescence was not observed after 30 min but was notable after 2 h of incubation. The fluorescence intensity of sfCherry increased gradually. Hence, using this method, the transcription and translation in giant lipid vesicles were observed (Fig. 4a).

Next, to investigate the effect of varying surface charges of giant lipid vesicles on the activation of transcription and translation, we prepared three types of giant lipid vesicles (DOPC, DOPC/DOPS [3:1 molar ratio], and DOPC/DOTAP [97:3 molar ratio]). The maximum median values of DFHBI-1T fluorescence in these three types of vesicles were observed at 30 min, which decreased gradually after 30 min. When 20 ng/μL (final concentration) of pDNA was encapsulated into the three types of giant lipid vesicles, despite the same encapsulation concentration, the DFHBI-1T fluorescence intensity in 3-mol%-DOTAP-containing vesicles was the highest compared with that of the other two types of giant lipid vesicles (Fig. 4b,c, Fig. S4a,b), possibly because the transcription was activated by the positively charged lipids. The DFHBI-1T fluorescence intensities in all the giant lipid vesicles decreased gradually (Fig. 4). Regarding translational activity, sfCherry fluorescence was detected approximately after 1 h incubation. The sfCherry fluorescence in 3-mol%-DOTAP-containing vesicles was the highest compared with that of the other two types of giant lipid vesicles (Fig. 4b,c, Fig. S4c,d). The translational activity in the DOPC vesicle or negatively charged lipid vesicles did not change. Similarly, the transcriptional activity in the same vesicles did not change. There is no association between the vesicle areas and the amount of transcription and translation (Fig. S5a–c). The fluorescence intensity of sfCherry was dependent on the fluorescence intensity of DFHBI-1T, indicating that the translational activity depended on the activation of transcription. The results showed that transcriptional and translational activities in the 3-mol% DOTAP-containing vesicles were the highest among those in the DOPC- or DOPS-containing vesicles (Fig. S4a–d).

**Figure 5:**
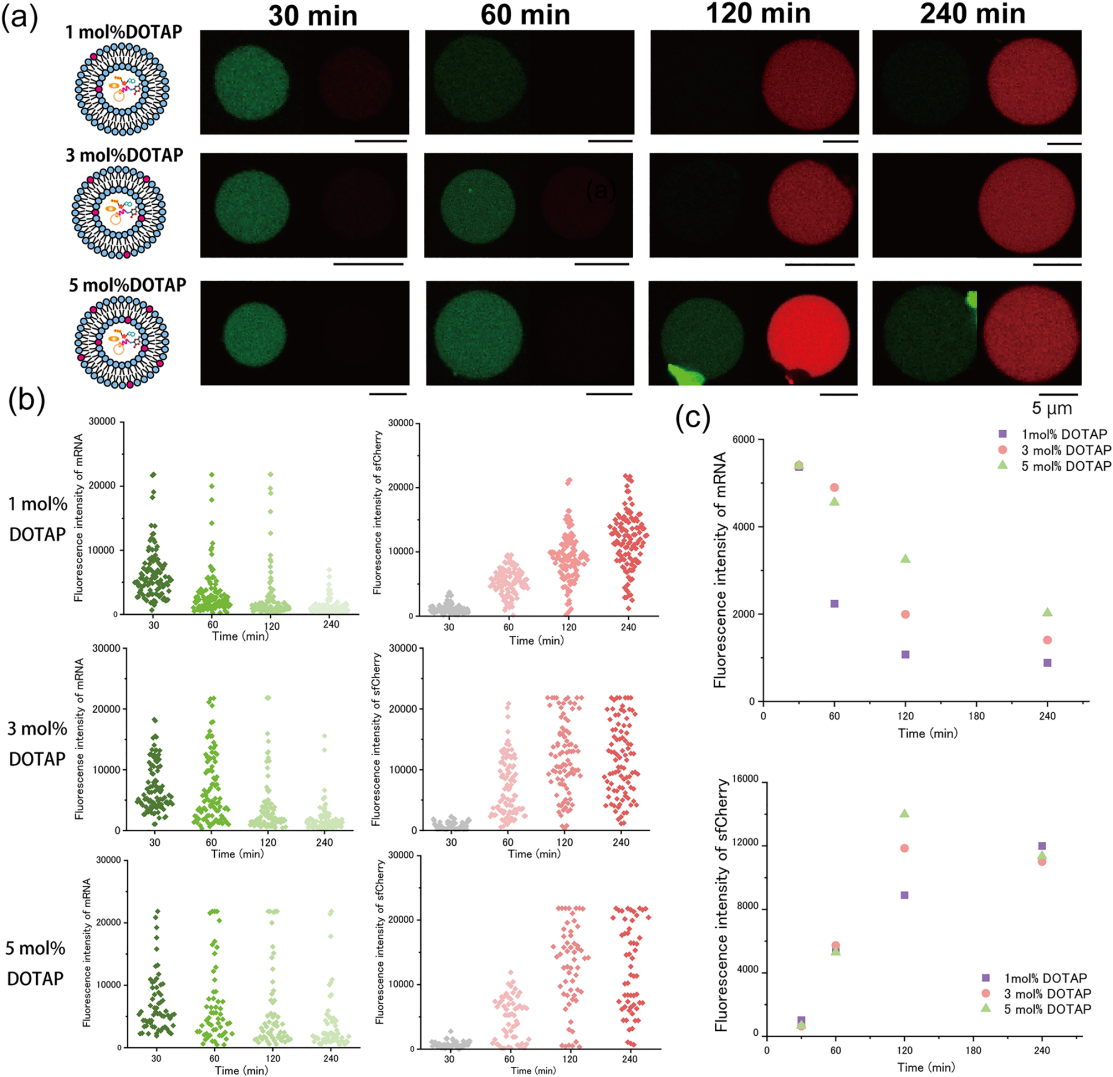
Comparison of transcription and translation inside giant lipid vesicles changing the concentration of cationic lipids. (**a**) Typical confocal images of the fluorescence intensity change inside the lipid vesicles containing DOPC/DOTAP (99:1 molar ratio), DOPC/DOTAP (98:2 molar ratio), and DOPC/DOTAP (97:3 molar ratio) on both leaflets. (**b**) DFHBI-1T fluorescence inside GUVs with the 1-mol%-DOTAP, 3-mol%-DOTAP, or 5-mol%-DOTAP membrane in response to time changes is shown in the left (green). sfCherry fluorescence of GUVs with 1-mol%-DOTAP, 3-mol%-DOTAP, or 5-mol%-DOTAP membrane in response to time changes is shown in the right (red). 1-mol%-DOTAP vesicles; 30 min (n = 111 (111 vesicles), N = 3 (three independent experiments), 60 min (n = 108, N = 3), 120 min (n = 116, N = 3), and 240 min (n = 118, N = 3). 3-mol%-DOTAP vesicles; 30 min (n = 104, N = 5), 60 min (n = 102, N = 5), 120 min (n = 90, N = 5), and 240 min (n = 92, N = 5). 5-mol%-DOTAP vesicles; 30 min (n = 64, N = 3), 60 min (n = 57, N = 3), 120 min (n = 58, N = 3), and 240 min (n = 57, N = 3). (c) Time-dependent changes in the fluorescence intensity of DFHBI-1T bound to messenger RNAs in the lipid vesicles. Comparison of the median value of the 1-mol%-DOTAP-containing lipid vesicle (purple), 3-mol%-DOTAP-containing lipid vesicle (pink), and 5-mol%-DOTAP-containing lipid vesicle (green). Time-dependent changes in the fluorescence intensity of sfCherry in the lipid vesicles. Comparison of the median value of the 1-mol%-DOTAP-containing lipid vesicle (purple), 3-mol%-DOTAP-containing lipid vesicle (pink), and 5-mol%-DOTAP-containing lipid vesicle (green).

Moreover, we investigated changes in transcriptional and translational activities in the vesicles by changing DOTAP concentrations (1 mol% and 5 mol%) (Fig. 5a). When the DOTAP concentration increased from 3 to 5 mol%, the decrease in the amount of synthesized mRNA in the 5-mol%-DOTAP-containing vesicles was slower than that in the 3-mol%-DOTAP-containing vesicles. After incubating for 120 min, the synthesized amount of sfCherry in the 5-mol%-DOTAP-containing vesicles was higher than that in the 3-mol%-DOTAP-containing vesicles (Fig. 5b,c). Conversely, when the DOTAP concentration decreased from 3 to 1 mol%, the decrease in the amount of synthesized mRNA in the 1-mol%-DOTAP-containing vesicles was faster than that in the 3-mol%-DOTAP-containing vesicles. After incubating for 120 min, the synthesized amount of sfCherry in the 1-mol%-DOTAP-containing vesicles was lower than that in the 3-mol%-DOTAP-containing vesicles (Fig. 5b,c). Moreover, transcriptional and translational activities in the 1-mol%-DOTAP-containing vesicles were the highest among those in the DOPC or DOPS-containing vesicles (Figs. 4c and 5c). The amounts of mRNA synthesized at 30 min and sfCherry synthesized at 240 min in the vesicles containing 1 mol%, 3 mol%, or 5 mol% DOTAP were almost the same (Fig. 5c). Thus, the presence of DOTAP in the vesicles increased the transcriptional and translational activities and played a role in preventing a digestion of mRNA (especially 5-mol%-DOTAP-containing vesicles).

**Figure 6:**
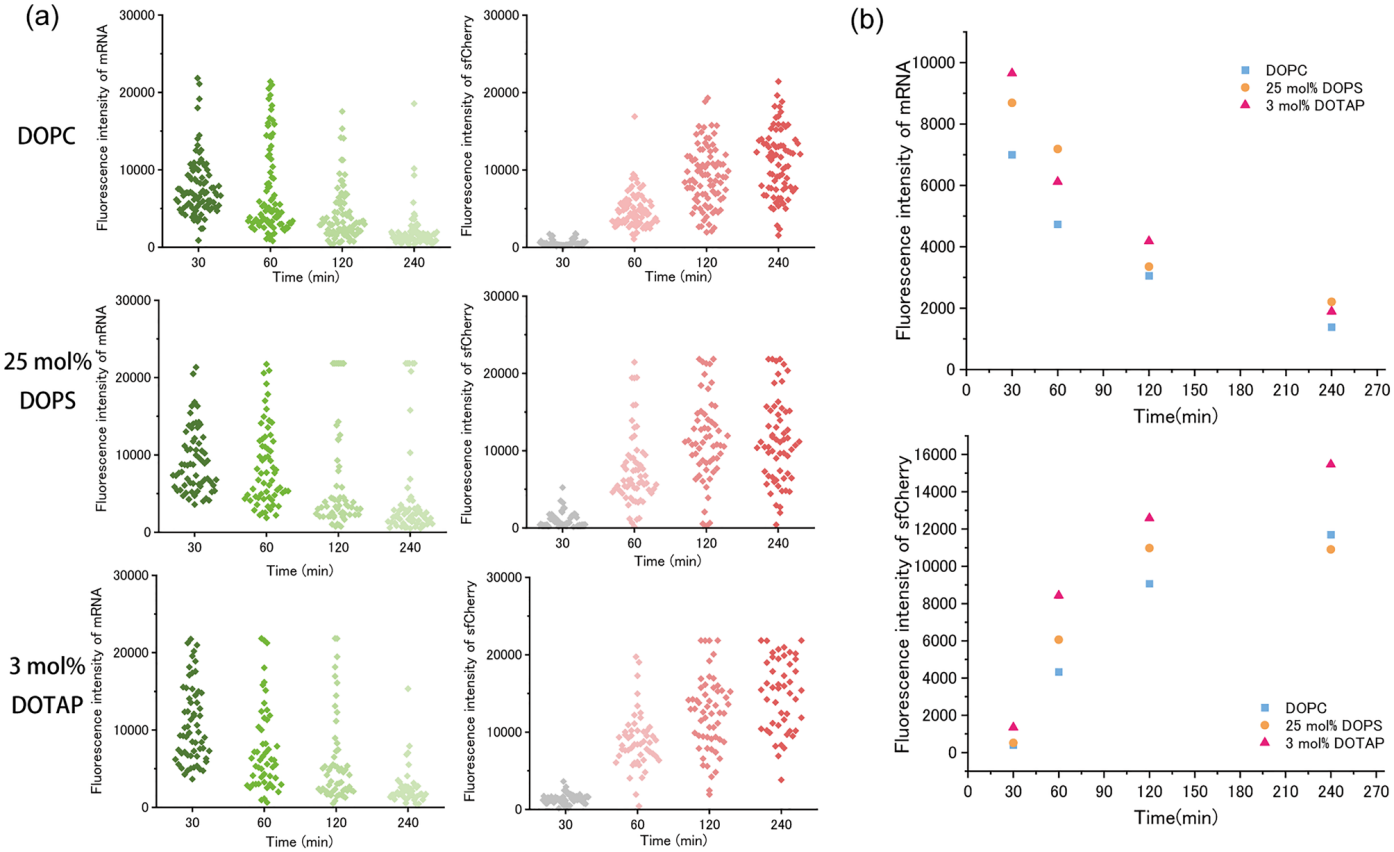
Comparison of transcriptional and translational activity under high plasmid DNA (pDNA) concentration condition (35 ng/μL of pDNA). (**a**) DFHBI-1T fluorescence inside GUVs with DOPC, 25-mol%-DOPS or 3-mol%-DOTAP membrane in response to time changing shown in left (green). SfCherry fluorescence of GUVs with DOPC, 25-mol%-DOPS or 3-mol%-DOTAP membrane in response to time changing shown in right (red). DOPC vesicles; 30 min (n = 92 (92 vesicles), N = 3 (three independent experiments)), 60 min (n = 83, N = 3), 120 min (n = 89, N = 3), and 240 min (n = 78, N = 3). 25-mol%-DOPS vesicles; 30 min (n = 66, N = 3), 60 min (n = 64, N = 3), 120 min (n = 58, N = 3), and 240 min (n = 60, N = 3). 3-mol%-DOTAP vesicles; 30 min (n = 61, N = 3), 60 min (n = 52, N = 3), 120 min (n = 57, N = 3), and 240 min (n = 47, N = 3). (**b**) Time-dependent changes in fluorescence intensity of DFHBI-1T bound to messenger RNA in the lipid vesicles. Comparison of the median value of DOPC-containing lipid vesicle (blue), 25-mol%-DOPS-containing lipid vesicle (orange), and 3-mol%-DOTAP-containing lipid vesicle (pink). Time-dependent changes in fluorescence intensity of sfCherry in the lipid vesicles. Comparison of the median value of DOPC-containing lipid vesicle (blue), 25-mol%-DOPS-containing lipid vesicle (orange), and 3-mol%-DOTAP-containing lipid vesicle (pink).

Next, we investigated the activations of the transcription and translation in pDNA-encapsulated giant lipid vesicles, with pDNA concentration changing from 20 to 35 ng/μL. The encapsulated pDNA varied among the three types of giant lipid vesicles when prepared with 35 ng/μL of pDNA (Fig. S3). The highest pDNA encapsulation concentration was in DOTAP-containing vesicles, followed by DOPC- and DOPS-containing vesicles. The DFHBI-1T fluorescence intensities in all types of giant lipid vesicles prepared with 35 ng/μL (approximately 9 nM) of pDNA were higher than those in the giant lipid vesicles prepared with 20 ng/μL (approximately 5 nM) of pDNA (Figs. 4b,c, 6a,b; Fig. S6a–c). Similarly, the fluorescence intensities of sfCherry in all types of giant lipid vesicles prepared with 35 ng/μL of pDNA were higher than those in the giant lipid vesicles prepared with 20 ng/μL of pDNA (Figs. 4b,c, 6a,b; Fig. S6d–f). In a previous study, the amounts of the synthesized mRNA and protein in the giant lipid vesicles containing pDNA concentrations of 1.75 nM and 3.5 nM were significantly different^30^. Thus, herein, changes in transcriptional and translational activities observed in the range of 5–9 nM of pDNA concentrations were reasonable. Among giant lipid vesicles prepared with 35 ng/μL pDNA, the DFHBI-1T fluorescence and sfCherry amount in DOTAP-containing vesicles were difficult to compare with the other two types of vesicles because the pDNA encapsulation concentration in DOTAP-containing vesicles was markedly high compared with that of other two types of vesicles (Fig. 2).

Additionally, to investigate the effect of transcriptional and translational activities attributed to the membrane surface charge, the zeta potential values of each vesicle composition were measured at a lipid vesicle concentration of approximately 1 mM, which was estimated by the molar concentration of the phospholipid in the inner leaflet of the lipid vesicle with a diameter of 10 μm. The zeta potential values of the vesicle membrane surface comprising DOPC, DOPC/DOPS [3:1 molar ratio], and DOPC/DOTAP [99:1 molar ratio, 97:3 molar ratio, and 95:5 molar ratio] hydrated by 10 mM HEPES/75 mM NaCl (pH 7.4) were − 3.18 ± 0.03 mV, − 42.0 ± 0.35 mV, − 2.49 ± 0.08 mV, − 0.71 ± 0.08 mV, and + 1.14 ± 0.39 mV, respectively (Fig. S7). Each zeta potential value of the lipid vesicles had the significant difference.

These results suggested that changes in transcriptional and translational activities depended on the lipid compositions of the giant lipid vesicles. The presence of positively charged lipids in high concentrations inhibited the transcriptional and translational activities when the protein was synthesized by the CFPS system outside the lipid vesicles^23,28^. In contrast, in this study, transcriptional and translational activities in the vesicles containing a low concentration (1–5 mol%) of positively charged lipids increased and 5-mol%-DOTAP-containing vesicles especially inhibited the digestion of mRNA. Although pDNA normally interacts with the membrane surface of vesicles containing positively charged lipids owing to electrostatic interactions, the present study showed that the pDNA did not interact with the positively charged vesicles containing the CFPS solution with the S30 extract (Fig. 2a, Fig. S1a). Electrostatic interactions did not occur between pDNA and the positively charged membrane owing to the presence of various biomolecules in the CFPS solution, implying that CFPS biomolecules were present in the lipid vesicles. Consequently, the transcriptional activity in the vesicles containing the positively charged lipid increased. Regarding the translation step, the ribosomal protein may be present in the inner phase of the vesicles containing positively charged lipids although the ribosomal protein with the positive charge and hydrophobicity easily interacted with the lipid membrane in the absence of positively charged lipids^23^. Consequently, the translational activity in the positively charged vesicles increased. The role of DOTAP (positively charged lipids) in protein synthesis using the CFPS system differed according to CFPS system conditions such as vesicle concentration in the reaction solution, charged lipid concentration in the vesicles, and CFPS types.

## Conclusion

In this study, using the cell-free protein system, we found that the changes in the transcription and translation inside the giant lipid vesicles were caused by changing the lipid composition of the giant lipid vesicles containing neutral-charged lipids, positively charged lipids, or negatively charged lipids. The transcriptional and translational activities in these vesicles containing CFPS systems were notably affected by the charge of phospholipids in giant lipid vesicles. The maximization of transcriptional and translational activities leads to highly efficient intracellular reactions in giant lipid vesicles. Therefore, the results of our study provide an experimental basis for constructing complex artificial cell models using the bottom-up approach, including biological reactions stimulated by the changes in the outer environment of the vesicles and the exchange of information between the living cells and lipid vesicles or lipid vesicles and lipid vesicles.

## Materials and methods

### Reagents

DOPC, DOPS, and DOTAP were purchased from Avanti Polar Lipids Inc. (Alabaster, AL, USA). Mineral Oil was purchased from Merck (Darmstadt, Germany). Glucose, sucrose, NaCl, and 2-(4-[2-hydroxyethyl]-1-piperazinyl)-ethanesulfonic acid (HEPES) were purchased from Wako Pure Chemical Industries, Ltd. (Tokyo, Japan). All aqueous solutions were prepared using ultrapure water obtained from a Milli-Q system (Milli-Q Integral 3; Merck KGaA, Darmstadt, Germany).

### Formation of giant lipid vesicles containing cell-free synthesis systems

The phospholipids DOPC, DOPC/DOPS (3:1 in a molar ratio), and DOPC/DOTAP (99:1, 97:3, and 95:5 in a molar ratio) were dissolved in chloroform and transferred into 4-mL glass tubes. The tubes were then placed in a vacuum desiccator for 1 h. A dry lipid film formed at the bottom of the glass tubes. Mineral oil was added to those films and the mixture was sonicated four times for 99 min at 50 °C and 44 W, and the final lipid concentration in mineral oil was l.1 mM. These lipid-in-oil solutions were stored at 4 °C in the dark and used within 2 weeks. To obtain a water-in-oil (w/o) emulsion including cell-free synthesis systems^34,35^, 1 × LMCPY, 0.175 mg/mL tRNA, 0.05% NaN_3_, 15 mM Mg (OAc)_2_, 1.5 mM each amino acid, 0.25 mg/mL creatine kinase, 67 ng/µL T7 RNA polymerase, 30% S30 extract, 40 µM DFHBI-1T dissolved into dimethyl sulfoxide, 20 ng/µL (or 35 ng/µL) plasmid containing sfCherry sequence and DFHBI-1T-binding sequence were added to 40 µL of lipid-in-oil solution in an Eppendorf tube and vortexed for 2 min. Both the components in the Eppendorf tube were emulsified by hand-tapping the tube. A thin lipid monolayer was formed at the interface between 30 µL of the lipid-in-oil solution and 30 µL of HEPES–glucose buffer (10 mM HEPES, 75 mM NaCl, and 500 mM glucose; pH 7.4). The w/o emulsion (45 µL) was added to the oil phase of the thin lipid monolayer. Finally, the solution was centrifuged to transfer the w/o emulsion through the lipid monolayer at 6900×*g*, 10 min^36^, 4 °C. The monolayer at the oil-buffer interface formed the outer leaflets of the giant lipid vesicles. After centrifugation, the giant lipid vesicles that had accumulated at the bottom of the tube were collected. The giant lipid vesicles were diluted by 20 µL of the outer solution (LMCPY, 29.88 µL; 5% NaN_3_, 0.8 µL; 1.6 M Mg[OAc]_2_, 0.744 µL; 20 mM each amino acid, 6.0 µL; S30 buffer, 24 µL; 5 mM DFHBI-1T dissolved into DMSO, 0.64 µL; and Milli-Q water, 17.92 µL).

### Estimation of the encapsulation concentration of pDNA in the giant lipid vesicles

A total of 3 µL of 1 mM YOYO-1 was added to 300 ng/µL sfCherry pDNA diluted in Milli-Q water and the mixture was incubated overnight at 4 °C. The sfCherry pDNA–YOYO-1 solution was dialyzed using 30 kDa cutoff membrane ultrafiltration (Amicon ultra-0.5) by washing twice with Milli-Q water to eliminate free YOYO-1. The giant lipid vesicles containing a solution of cell-free synthesis system and YOYO-1-conjugated pDNA were generated by the droplet transfer method in the same manner as described above. The giant lipid vesicles containing YOYO-1-conjugated pDNA were observed using a confocal laser scanning microscope (CLSM) (FV-3000, Olympus, Tokyo, Japan) with an oil-immersion lens (× 60) and using a diode laser (448 nm) for YOTO-1 (500–600 nm).

### Observation of transcription and translation of CFPS system in 384-well plate

Twenty µL of cell-free extract mixture was prepared on ice and quickly pipetted into a 384-well black plate. Fluorescence intensity of DHFBI-1T (Ex. 482 nm, Em. 505 nm) and sfCherry (Ex. 570 nm, Em. 620 nm) was monitored using a microplate reader (Synergy H1 Multimode Reader, Biotek, Winooski, VT, USA) every 5 min for 240 min at 37 °C.

### Observation of transcriptional and translational activity in the giant lipid vesicles

The giant lipid vesicle solution was separated into four tubes, which were incubated at 37 °C for 30 min, 1, 2, and 4 h. After incubation, the fluorescence in giant lipid vesicles was observed using a CLSM (FV-3000, Olympus, Tokyo, Japan) with an oil-immersion lens (× 60), using a diode laser (488 nm) for DFHBI-1T (500–540 nm), and a diode laser (561 nm) for sfCherry (570–670 nm) to assess the transcriptional and translational activity. The fluorescence intensities inside the giant lipid vesicles were measured using the ImageJ software.

### Measurement of zeta potential on the vesicle membrane surface

The lipid films (DOPC, DOPC/DOPS [7:3 in a molar ratio], and DOPC/DOTAP [99:1, 97:3, and 95:5 in a molar ratio]) were hydrated by adding 10 mM HEPES/75 mM NaCl (pH 7.4) and vortexed to prepare 1 mM liposomes. The zeta potential values of the 1 mg/mL nano-sized liposomes were measured using the Zetasizer nano ZPS instrument (Malvern Instruments, United Kingdom).

### Supplementary Information

Supplementary Figures.

## Data Availability

The datasets used and/or analyzed during the current study are available from the corresponding author on reasonable request.
