# Supplementary material for: Efficiency of transcription and translation of cell-free protein synthesis systems in cell-sized lipid vesicles with changing lipid composition determined by fluorescence measurements
Source: Sci Rep. 2024 Feb 3;14:2852. doi: 10.1038/s41598-024-53135-8 (PMC10838264; doi:10.1038/s41598-024-53135-8)
Supplement: Supplementary file 1 — Supplementary Figures. [file 41598_2024_53135_MOESM1_ESM.docx]

Supporting Information

**Efficiency of transcription and translation of cell-free protein synthesis systems in cell-sized lipid vesicles with changing lipid composition determined by fluorescence measurements**

Akari Miwa^1^ , Masatoshi Wakamori^2^ , Tetsuro Ariyoshi^3,4^ , Yasushi Okada^3,4,5^ , Mikako Shirouzu^6^ , Takashi Umehara^2^, and Koki Kamiya^1＊^

^1^Division of Molecular Science, Graduate School of Science and Technology, Gunma University, 1-5-1 Tenjin-cho, Kiryu, Gunma 376-8515, Japan

^2^Laboratory for Epigenetics Drug Discovery, RIKEN Center for Biosystems Dynamics Research, 1-7-22 Suehiro-cho, Tsurumi-ku, Yokohama 230-0045, Japan

^3^Laboratory for Cell Polarity Regulation, RIKEN Center for Biosystems Dynamics Research, 6-2-3 Furue-dai, Suita, Osaka 565-0874, Japan

^4^Department of Cell Biology, Graduate School of Medicine, and International Research Center for Neurointelligence (WPI-IRCN), the University of Tokyo, 7-3-1 Hongo, Bunkyo-ku, Tokyo 113-0033, Japan

^5^Department of Physics and Universal Biology Institute (UBI), Graduate School of Science, the University of Tokyo, 7-3-1 Hongo, Bunkyo-ku, Tokyo 113-0033, Japan

^6^Laboratory for Protein Functional and Structural Biology, RIKEN Center for Biosystems Dynamics Research, 1-7-22 Suehiro-cho, Tsurumi-ku, Yokohama 230-0045, Japan

＊Corresponding author: K. K

Tel: (+81)-277-30-1342; Fax: (+81)-277-30-1342; E-mail: kamiya@gunma-u.ac.jp

**Fig.S1** (a) Typical confocal images of the fluorescence intensities of oxazole yellow homodimer (YOYO-1)-conjugated sfCherry-producing plasmids under each membrane condition. (b) Calibration curve for yoyo-1-plasmid DNA concentration *vs.* Fluorescence intensity. Error bars indicate mean±SD. (c) Distribution of DNA concentration estimates (ng/µL) inside GUVs using DOPC under the 20 ng/µL YOYO-1-plusmid DNA condition (n=121, three independent experiments). (d) Distribution of DNA concentration estimates (ng/µL) inside GUVs using 25 mol% DOPS under the 20 ng/µL YOYO-1-plusmid DNA condition (n=90, three independent experiments). (e) Distribution of DNA concentration estimates (ng/µL) inside GUVs using 3 mol% DOTAP under the 20 ng/µL YOYO-1-plusmid DNA condition (n=86, three independent experiments). (f) Distribution of DNA concentration estimates (ng/µL) inside GUVs using 1 mol% DOTAP under the 20 ng/µL YOYO-1-plusmid DNA condition (n=89, three independent experiments). (g) Distribution of DNA concentration estimates (ng/µL) inside GUVs using 5 mol% DOTAP under the 20 ng/µL YOYO-1-plusmid DNA condition (n=105, three independent experiments). (h) Distribution of DNA concentration estimates (ng/µL) inside GUVs using DOPC under the 35 ng/µL YOYO-1-plusmid DNA condition (n=123, three independent experiments). (i) Distribution of DNA concentration estimates (ng/µL) inside GUVs using 25 mol% DOPS under the 35 ng/µL YOYO-1-plusmid DNA condition (n=65, three independent experiments). (j) Distribution of DNA concentration estimates (ng/µL) inside GUVs using 3 mol% DOTAP under the 35 ng/µL YOYO-1-plusmid DNA condition (n=84, three independent experiments).


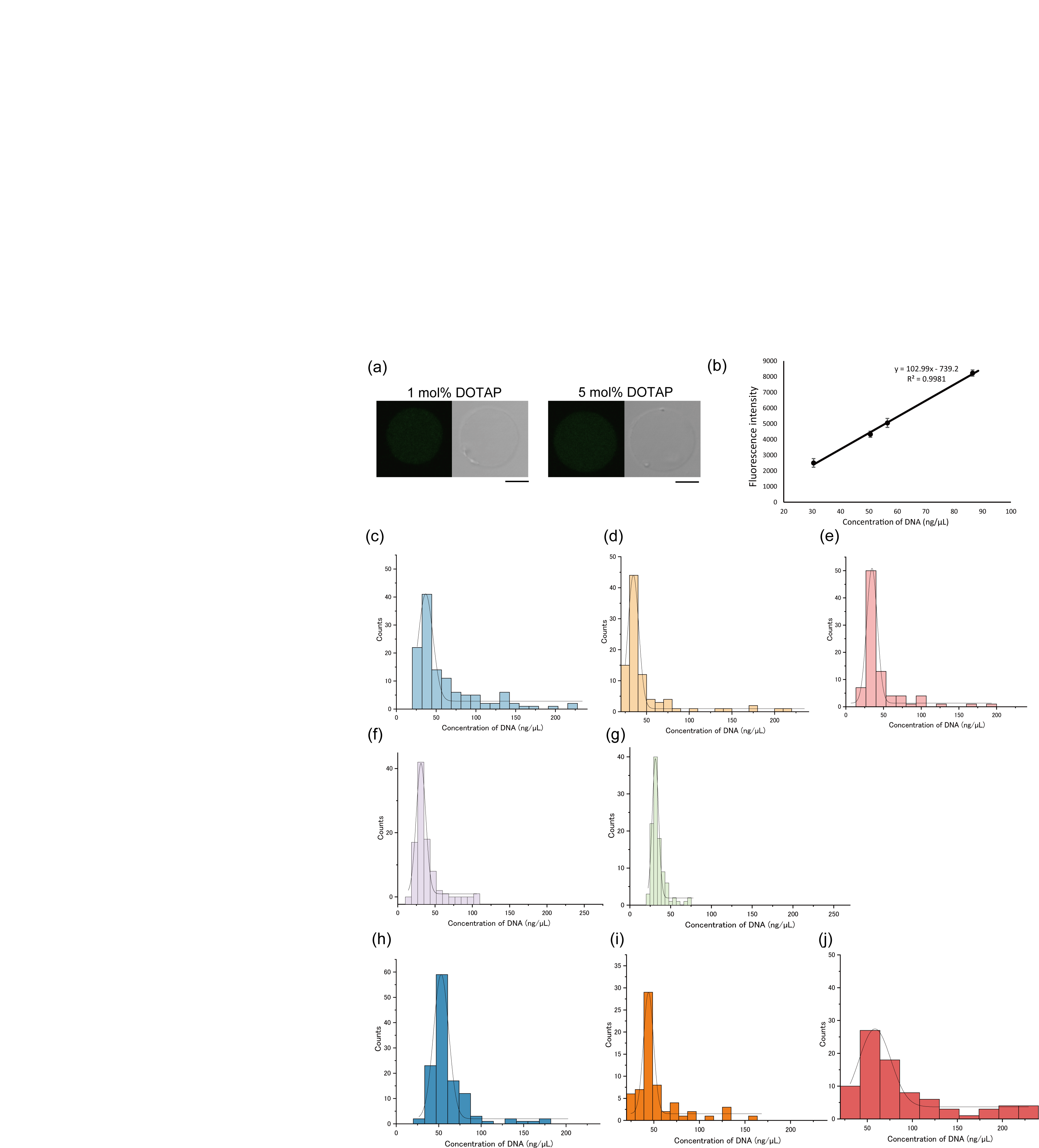


**
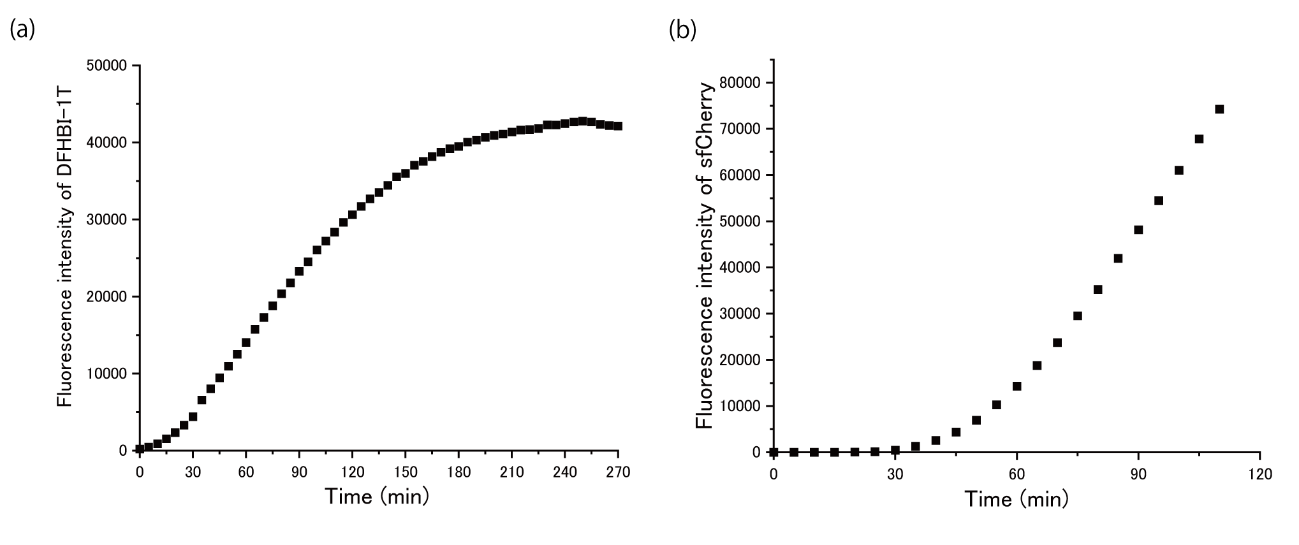
**

**Fig.S2** Time-dependent change of Fluorescence intensity of DFHBI-1T and sfCherry using PURE system. (a)Mean values (two independent experiments) of fluorescence intensity of DFHBI-1T for 270 min at 37℃. (b)Mean values (two independent experiments) of fluorescence intensity of sfcherry for 110 min at 37℃.


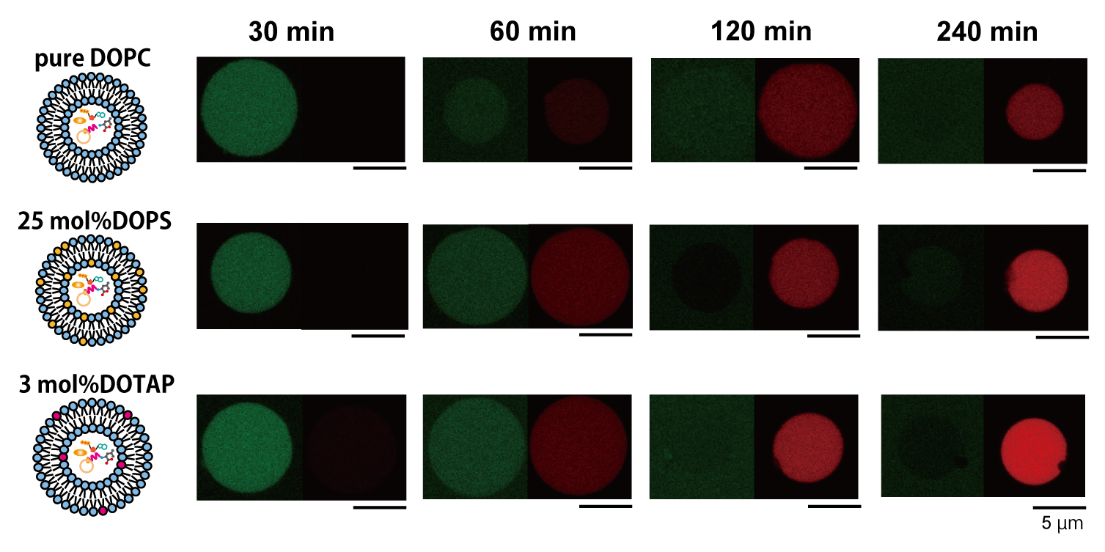


**Fig.S3** Typical confocal images of the fluorescence intensity change inside the lipid vesicles containing DOPC, DOPC/DOPS (75:25 molar ratio), or DOPC/DOTAP (97:3 molar ratio) on both leaflets under the 35 ng/µL pDNA concentration.


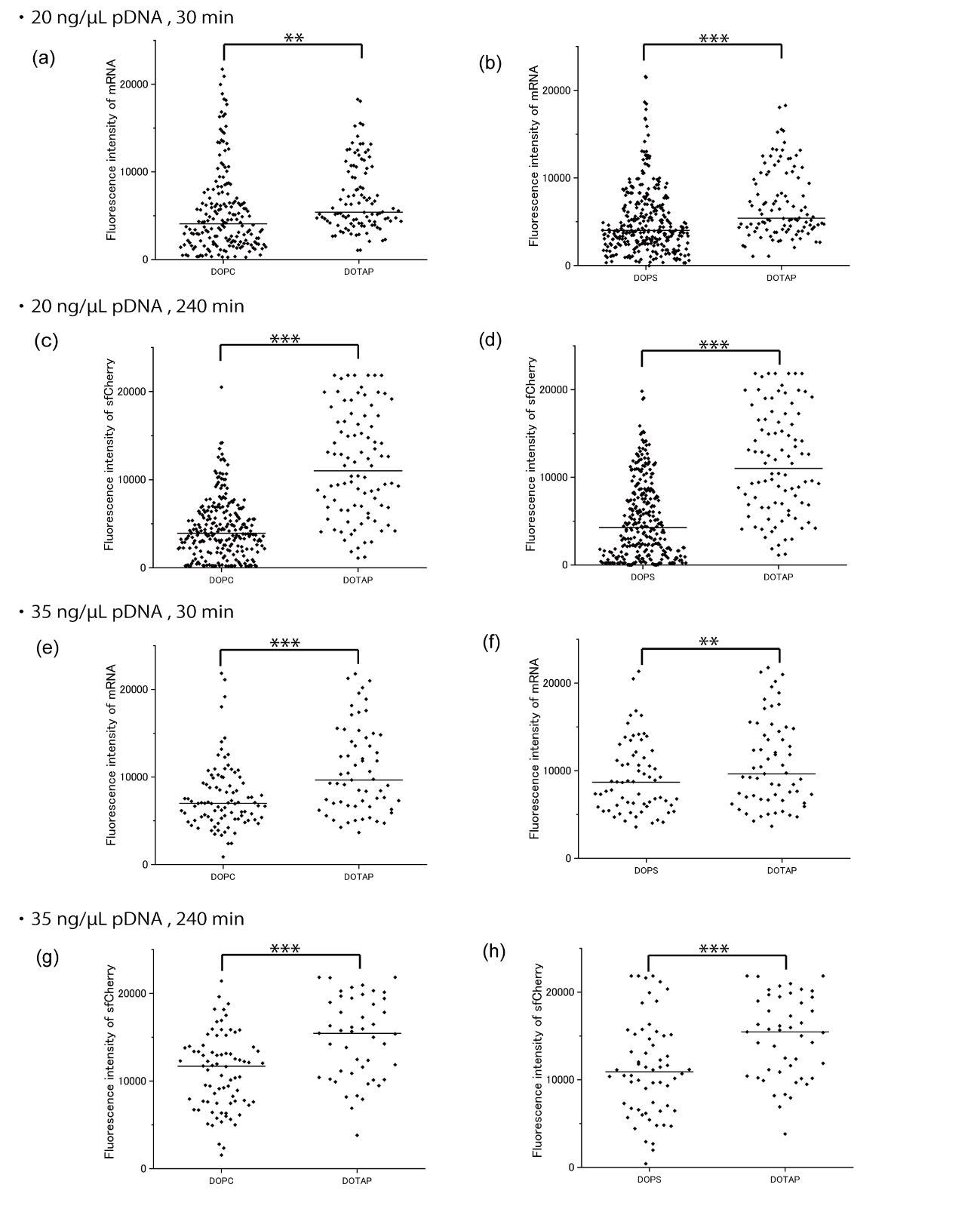


**Fig.S4** (a, b, e and f) Comparison results of the fluorescence intensity of mRNA at 30min between DOPC/DOTAP (97:3 molar ratio) and DOPC or DOPC/DOPS (75:25 molar ratio) by t-test analysis. (c, d, g and h) Comparison results of the fluorescence intensity of sfCherry at 240min between 3% DOTAP and DOPC or 25% DOPS by t-test analysis. ** t-test *P* value <0.01. *** t-test *P* value <0.001.


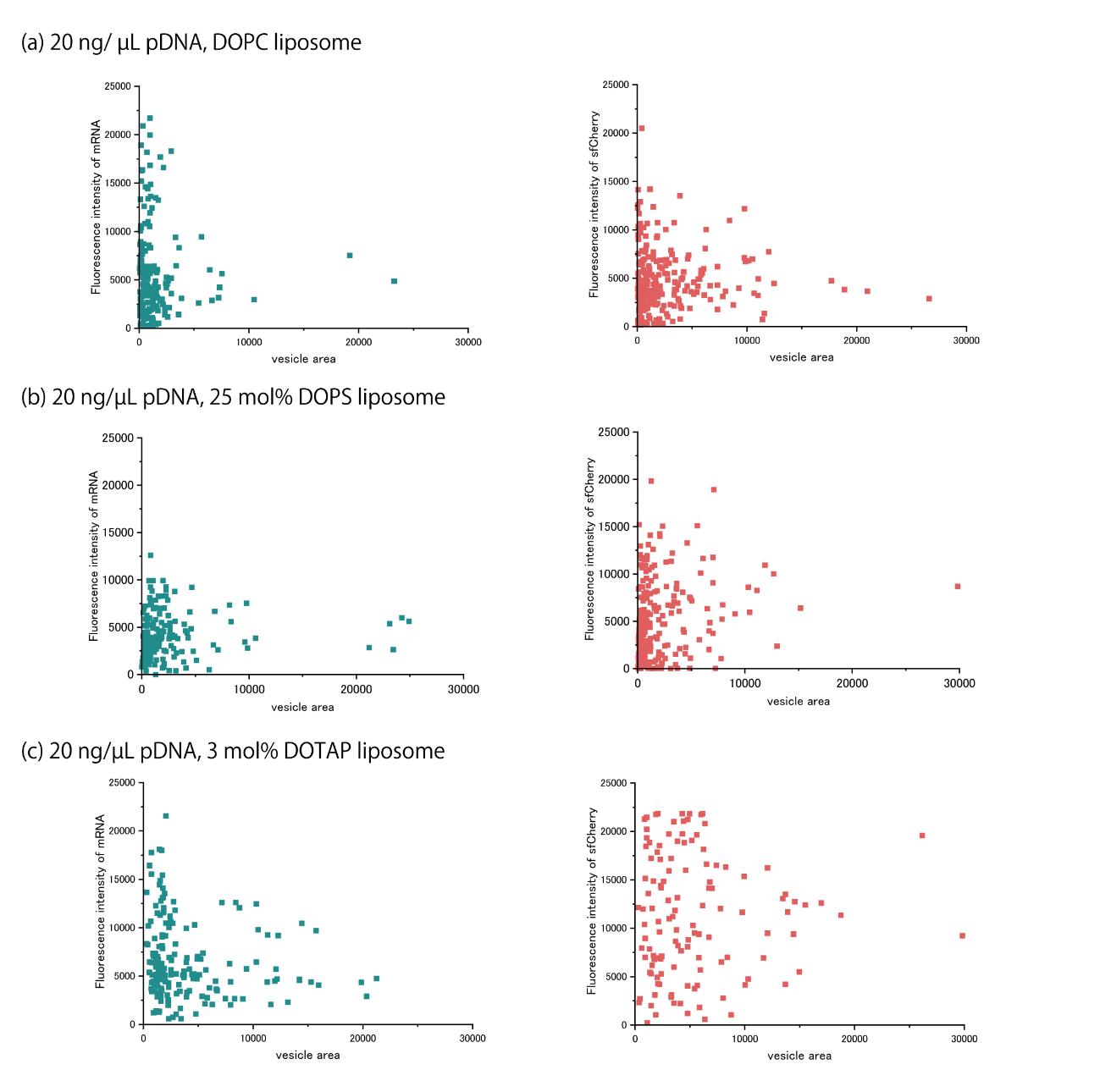


**Fig.S5** Scatter plot distribution of the fluorescence intensity of mRNA (DFHBI-1T) or sfCherry *vs.* the vesicle area. (a) Data represented the fluorescence intensity of mRNA at 30 min and vesicle area (n=184, two independent experiments), the fluorescence intensity of sfCherry at 240 min and vesicle area (n=247, two independent experiments). (b) Data represented the fluorescence intensity of mRNA at 30 min and vesicle area (n=318, three independent experiments), the fluorescence intensity of sfCherry at 240 min and vesicle area (n=307, three independent experiments). (c) Data represented the fluorescence intensity of mRNA at 30 min and vesicle area (n=173, five independent experiments), the fluorescence intensity of sfCherry at 240 min and vesicle area (n=123, five independent experiments).


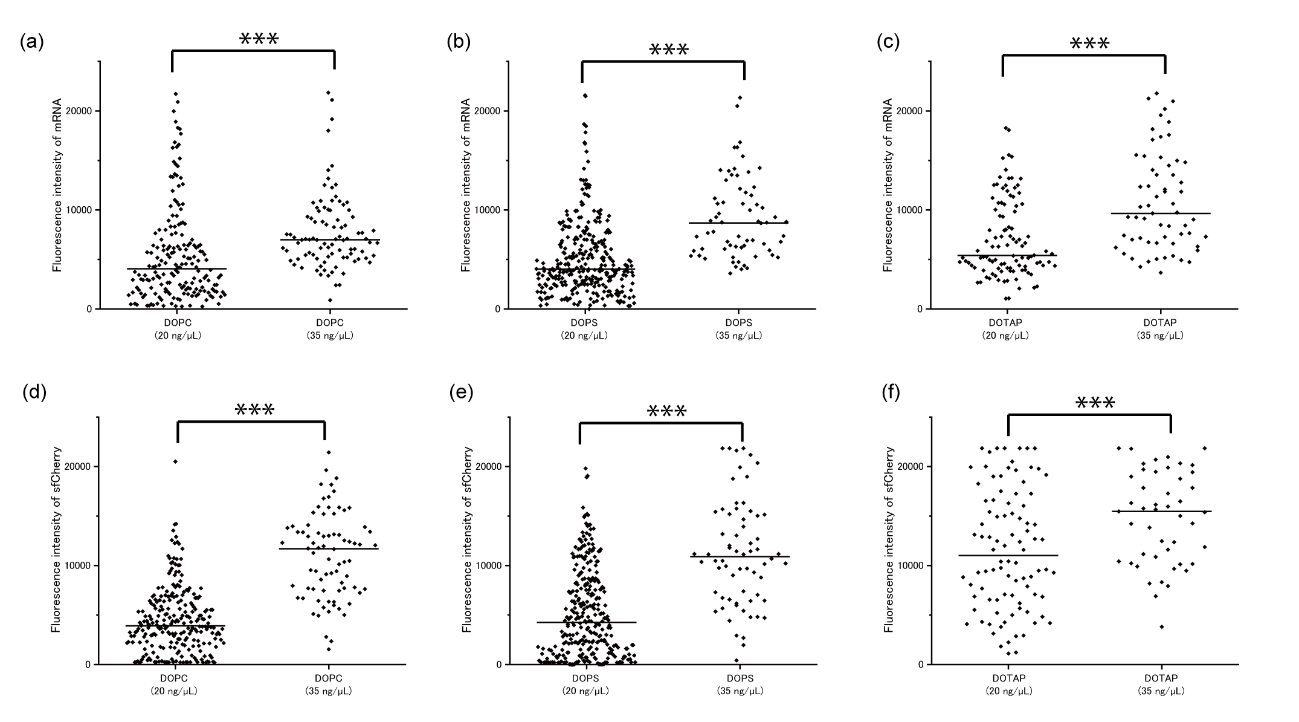


**Fig.S6** (a, b and c) Comparison results of the fluorescence intensity of mRNA at 30min between the 20 ng/µL pDNA condition and the 35 ng/µL pDNA condition by t-test analysis. (d, e and f) Comparison results of the fluorescence intensity of sfCherry at 240min between the 20 ng/µL pDNA condition and the 35 ng/µL pDNA condition by t-test analysis. *** t-test *P* value <0.001.


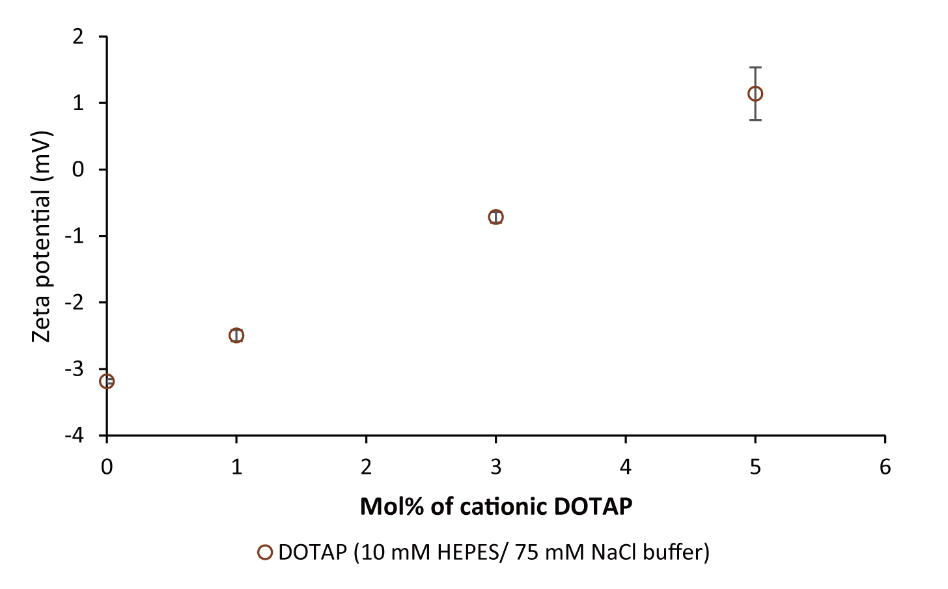


**Fig.S7** The change in zeta potential with increasing concentration (mol%) of cationic lipid (DOTAP). Data are expressed as the mean ± SD, n =3.
